# Oral microbiome alterations and their association with long-term heavy metal exposure and early health effects

**DOI:** 10.1080/20002297.2026.2647511

**Published:** 2026-03-23

**Authors:** Hongling Liu, Jia Li, Keke Yang, Huan Li, Susu Cao, Yuanyuan Bao, Lu Feng, Li Zhang, Jingping Niu, Tian Tian

**Affiliations:** a School of Public Health, Lanzhou University, Lanzhou, Gansu, The People's Republic of China; b School of Public Health, Fudan University, Shanghai, The People's Republic of China; c School of Stomatology, Lanzhou University, Lanzhou, Gansu, The People's Republic of China

**Keywords:** Heavy metals, biomarkers, internal exposure, oral bacteria, metagenomic sequencing, early health effects, KEGG

## Abstract

**Background:**

Long-term heavy metal exposure poses health risks, and non-invasive biomarkers for early detection are needed.

**Objective:**

This study investigated whether oral microbiome alterations can serve as a non-invasive indicator of long-term HMs exposure and associated early biological effects.

**Design:**

Soil, buccal mucosa, blood, and urine samples were collected from contaminated (CA) and uncontaminated (UA) areas. Soil contamination was assessed, and internal biomarkers were measured. Oral bacterial diversity was analyzed using metagenomic sequencing.

**Results:**

Severe Cd and Pb contamination was found in CA soil. Participants in CA had elevated internal Cd levels, renal impairment, and immune alterations. Oral microbiome analysis revealed decreased alpha diversity, reduced network complexity, and a shift from beneficial to pathogenic keystone taxa in CA. Functional analysis showed enrichment of stress-response pathways, suppression of metabolic pathways, and increased pathways linked to human diseases. Specific bacterial taxa correlated with internal biomarker levels.

**Conclusions:**

There is a close association between long-term HMs exposure and reproducible, multi-faceted shifts in the oral microbiome. The oral microbiome may represent a promising, non-invasive biomarker for assessing environmental exposure and its early biological impacts.

## Introduction

The mining and smelting of non-ferrous metals are among the important pillar industries of China [1]. However, a number of HMs are generated during its beneficiation and tailings discharge processes. Through pathways such as atmospheric deposition and water transport, these HMs contaminate soil [2,3]. Soil HMs contamination is characterised as insidious, cumulative and persistent [4]. It poses a dual threat: not only depleting soil resources but also entering the human body via the soil‒crop‒food chain, where biomagnification leads to their accumulation [5]. HMs exposure can not only directly cause disease and death in humans. This can also lead to imbalances in microbial homoeostasis by altering the structure and composition of human microbial communities, which can further contribute to a wide range of diseases and potential risks to human health [6].

Biomarkers are measurable indicators that reflect the internal burden of a pollutant and the associated physiological, biochemical and organ function changes it triggers. Internal exposure biomarkers and biomarkers of biological effects are commonly used to assess the levels of environmental HMs exposures in the human body and the biological effects they produce. Blood Cd is typically used to evaluate near-term exposure, whereas urine Cd is utilised to assess systemic load and long-term exposure [7]. For instance, long-term exposure to nephrotoxic HMs, such as Cd, leads to a reduced glomerular filtration rate and altered levels of other biomarkers of kidney injury [8]. HMs exposure has been shown to cause significant increases in urinary indicators such as β_2_-MG and 8-OHG [9,10]. Furthermore, inflammatory effect markers such as the systemic immune-inflammation index (SII) and pro-inflammatory factors are significantly correlated with HMs exposure [11]. Specific correlations have been observed, including between blood Cd and cytokines (IL-1β, IL-6 and IL-8), and between urinary biomarkers (8-OHG and β2-MG) and HMs such as Cd and Pb [10]. Thus, biomarkers provide a critical approach for assessing internal HMs doses and elucidating their health effects.

The oral cavity is an organ that is directly exposed to the outside world. Its unique anatomy and physiology provide a highly heterogeneous ecological niche for microbial colonisation [12]. The resident microbial community is highly diverse, encompassing bacteria, fungi, viruses and protozoa. This complexity makes it the second-largest microbiome in the human body, after the gut microbiome [13]. The oral microbiome plays a significant role in maintaining host health [14]. An imbalance in oral microorganisms not only leads to oral diseases, but is also associated with the development of systemic diseases [14]. In recent years, the impact of HMs exposure on the oral microbiome has garnered increasing research attention because of increasing environmental pollution [15]. Epidemiologic studies have confirmed that the levels of the HMs Sb, As and Hg can significantly alter the oral bacterial composition, posing a potential risk to oral health. Pb exposure leads to an increase in oral antibiotic-resistant bacteria [6]. It has also been shown that co-exposure of *Streptococcus mitis* and Ni increases the chances of sensitisation in host individuals [16]. Despite these insights, the comprehensive effects of HMs exposure on the human oral microbiome remain insufficiently understood.

The study site for this research is located in Baiyin City, Gansu Province, one of the most important multi-species non-ferrous metal industrial bases in China [17]. Elevated levels of HMs are present in the local soil, air and surface water, indicating severe ecological and potential early biological effects [18]. In addition, owing to the lack of water resources, the local participants have a long-standing habit of irrigating their agricultural lands with domestic and industrial wastewater, which further increases the accumulation of HMs in the local agricultural soils [19]. This practice likely contributed to the accumulation of HMs in the local bodies of participants. The UA was selected from Xinglongshan Nature Reserve of Lanzhou City, Gansu Province, which has similar geographic and climatic conditions to Baiyin City. The Xinglongshan Nature Reserve is almost free from HMs pollution. By comparing the buccal mucosa microbiome between participants from these two areas, this study characterised the alterations associated with long-term environmental HMs exposure. The aims of this study were to (a) assess the level of environmental HMs contamination by detecting HMs concentrations in soil; (b) investigate the changes in human biomarkers linked to long-term environmental HMs exposure; (c) analyse the associated changes in the community structure, genetic diversity, and functional pathways of the oral microbiota using metagenomic sequencing; and (d) explore the potential of oral microorganisms as biomarkers of exposure. This work seeks to provide a more systematic understanding of how HMs exposure is related to human health, specifically by addressing the critical knowledge gap regarding the oral microbiome's response to chronic environmental HMs exposure.

## Materials and methods

### Selection of study areas

Long-term metal mining and smelting activities have resulted in severe environmental HMs pollution. Accordingly, Baiyin City (104°17′38.4972″E, 36°29′6.2844″N) was selected as the contaminated area (CA). The uncontaminated area (UA) was Lanzhou City (104°1′8.6736″E, 35°46′5.8332″N), which is located approximately 100 km upwind from Baiyin and has no history of relevant industrial pollution. The two cities are comparable in terms of geography, climate, and socioeconomic status, thereby helping to control for these macro-level confounders.

### Participant recruitment and characteristics

This study employed a cross-sectional design to compare the internal HMs burden, associated biomarkers, and oral microbiome composition between long-term participants with CA and UA. Participants were recruited from two adjacent villages in each area between September 2019 and January 2021. The following inclusion and exclusion criteria were applied to control for potential confounders. Inclusion criteria: aged 40–69 years and had permanent residency for ≥10 years. Exclusion criteria: aged 40–69 years and had been permanent participants for at least 10 years. Those who had used antibiotics within the previous 3 months, had immune system disorders, or worked in HMs-related occupations (e.g. mining/smelting) were excluded. A total of 308 participants were enroled: 183 from the CA and 125 from the UA. All provided blood samples, and 297 participants (CA: *n* = 180; UA: *n* = 117) provided valid first-morning urine samples that met quality control standards. As an exploratory survey aiming to generate hypotheses in a previously understudied population, a formal a priori sample size calculation was not feasible due to the lack of prior data on effect sizes. Therefore, the sample size was based on all eligible and consenting individuals available during the study period, which is consistent with the design of a community-based exploratory survey.

Detailed demographic and lifestyle data (age, sex, height, weight, occupation, smoking and alcohol consumption) were collected via questionnaires. We defined the key lifestyle factors as follows. Smoking status was categorised as current smoker (smoking daily at the time of survey) or non-smoker. Alcohol consumption was assessed by frequency and categorised into three groups: never drinkers (lifetime consumption <12 times), occasional drinkers (less than once per month), and regular drinkers (at least once per month). For statistical adjustment in the primary analyses, alcohol use was simplified to a binary variable: regular drinker (≥1 time/month) versus non-regular drinker. The comparability of the CA and UA groups in terms of these variables was assessed (Table 1), and no significant differences were found in key demographic factors. These variables were treated as covariates in subsequent statistical models to control for potential confounding. To specifically investigate the association between HMs exposure and the oral microbiome while minimising major confounders, a sub-cohort was selected for buccal mucosa sampling. Initial eligibility for this sub-cohort required participants to be non-smokers and non-drinkers and to have good oral health. The detailed, multi-step selection process, including subsequent molecular and sequencing quality control that determined the final sample size, is described in Section 2.5.

**Table 1.** Concentrations of heavy metals in soil from uncontaminated (UA) and contaminated (CA) areas.

Elemental mg/kg	UA (*n* = 36)	CA (*n* = 44)	Background value	*P-*value
Mo	0.588 (0.517, 0.678)	0.462 (0.378, 0.619)	0.920	0.010
Sb	0.925 (0.836, 1.021)	1.547 (0.792, 2.723)	1.140	<0.001
Pb	13.613 (12.146, 14.962)	70.956 (22.704, 158.576)	22.900	<0.001
Mn	398.500 (360.436, 454.033)	304.834 (274.220, 344.499)	694.000	<0.001
Co	6.890 (6.220, 8.218)	4.759 (4.225, 5.231)	14.600	<0.001
Cu	16.811 (12.310, 35.350)	34.866 (15.665, 70.749)	30.500	0.017
Zn	53.746 (42.771, 59.167)	117.849 (59.154, 462.924)	72.700	<0.001
Cd	0.162 (0.146, 0.197)	2.944 (0.903, 10.428)	0.180	<0.001

Note: Soil heavy metal concentrations are presented as median and interquartile range (IQR; Q1, Q3). Differences for each heavy metal were assessed using the Mann‒Whitney U test. *P* < 0.05 is considered statistically different.

The study protocol was approved by the Ethics Committee of the School of Public Health, Lanzhou University, and written informed consent was obtained from all participants.

### Collection of soil samples

**Figure 1.**
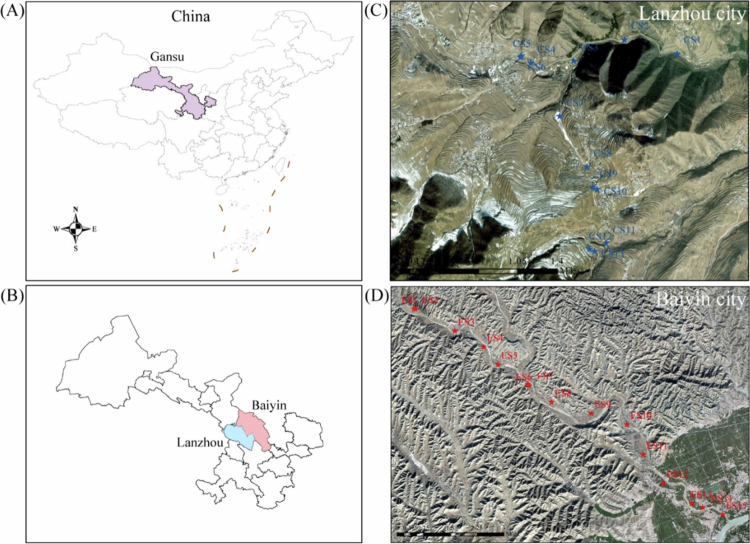
Geographic location of the study area and sampling sites. The study was conducted in Gansu Province, China, comparing a heavy metal contaminated area (CA) in Baiyin City with an uncontaminated control area (UA) in Lanzhou City. The left panel provides the geographical context: (A) China, with Gansu Province highlighted, and (B) Gansu Province, showing the locations of the two cities. The right panel shows the sampling sites within each city: (C) UA sites in Lanzhou (blue points) and (D) CA sites in Baiyin (red points). This map establishes the distinct environmental settings that define the two comparison groups in this study.

A total of 28 agricultural soil sampling points were established: 15 in the CA and 13 in the UA (Figure 1C,D). At each sampling site, five subsampling sites were set up using a five-point sampling method in a randomly selected area of approximately 10 × 10 m in the agricultural field. The soil was collected using a sterile wooden spatula at a depth of approximately 20 cm at five subsampling sites and mixed into one sample. Three parallel samples were collected from each subsampling site, and a total of 84 soil samples were collected. The samples were sealed in polyethylene bags, transported to the laboratory within 24 h and stored at 4 °C for analysis of the soil HMs concentrations. HMs (Mo, Sb, Pb, Mn, Co, Cu, Zn and Cd) concentrations were determined using inductively coupled plasma‒mass spectrometry (ICP‒MS, Agilent, USA). The soil samples were allowed to dry naturally at room temperature, and stones, roots, and other impurities were removed through a 200-mesh nylon sieve. This step was followed by a microwave digestion system (Sartorius, PB-10, Germany) using approximately 0.05 g of each sample. Failed samples were excluded, and a total of 80 soil samples were used for subsequent analysis.

### Collection of blood and urine samples

Peripheral blood and first-morning urine were collected from the participants. To study the early biological effects associated with HMs, blood Cd and urine Cd concentrations were determined using ICP–MS. For blood, 1 ml of whole blood was aliquoted equally into two 2 ml cryovials for the determination of blood Cd. The remaining blood was centrifuged at 1,000  r/min for 10 min to obtain plasma, which was used to analyse the levels of IL-2, IL-4, IL-6, IL-8 and TNF-*α*. SII was used as a biomarker of inflammatory bioeffects to respond to the immune status of the organism and was calculated as follows [11]: SII = neutrophil count/lymphocyte count × platelet count.

Morning urine samples were collected using containers that were soaked in dilute HNO_3_ for 24  h before use and rinsed well with deionized water to remove possible Cd contamination. A total of 16  ml of the 1st mid-morning urine of the investigated subjects was collected, and 5 ml of urine was acidified with concentrated HNO₃ to a pH < 2 prior to Cd analysis. Urine biomarkers (β_2_-MG, 8-OHG) were corrected for specific gravity to account for dilution. A total of 5 ml of each urine samples were taken and added to 1 mol/L NaOH to bring the pH to approximately 8 for β_2_-MG determination. The remaining urine was used for 8-OHG determination.

### The collection of buccal mucosa samples

To minimise the confounding effects of lifestyle and oral health on the microbiome, a sub-cohort was selected for buccal mucosa sampling. All participants met the following eligibility criteria: (1) The resident has no history of smoking and no habit of drinking alcohol; (2) Participants did not have oral diseases such as halitosis, chronic dry mouth, untreated decayed tooth lesions, abscesses, cancer, or candidiasis; (3) Participants maintained stable residence in the local area and did not work outside the home for more than half a year consecutively; and (4) Participants possessed at least 24 teeth. Buccal mucosa samples were collected from this sub-cohort prior to breakfast, after which the participants rinsed their mouths with deionized water for 30 min. A trained professional collected three replicate swabs from the right and left buccal cavities of each participant using sterile cotton swabs to ensure sufficient biomass. The samples were immediately transported and stored at −80 °C. All collected samples subsequently underwent DNA extraction, library preparation, and metagenomic sequencing (detailed in Section 2.6). Samples that failed to meet predefined quality control thresholds were excluded. After applying all eligibility and quality filters, a total of 37 high-quality buccal mucosa samples constituted the final analytical cohort (CA: *n* = 26; UA: *n* = 11).

### Metagenomic DNA isolation, sequencing and analysis

The total genomic DNA was extracted in strict accordance with the instructions of the DNA extraction kit. The concentration and purity of the extracted DNA were determined with SynergyHTX and NanoDrop2000, respectively. DNA quality was checked on a 1% agarose gel.

DNA extract was fragmented to an average size of approximately 400 bp using Covaris M220 (Gene Company Limited, China) for paired-end library construction. A paired-end library was constructed using NEXTFLEX Rapid DNA-Seq (Bioo Scientific, Austin, TX, USA). Paired-end sequencing was performed on an Illumina NovaSeq™ X Plus (Illumina Inc., San Diego, CA, USA) at Majorbio Bio-Pharm Technology Co., Ltd. (Shanghai, China) using NovaSeq X Series 25B Reagent Kit according to the manufacturer’s instructions (www.illumina.com).

The data were analysed on the free online platform of Majorbio Cloud Platform (www.majorbio.com). Briefly, the raw sequencing reads were trimmed of adaptors, and low-quality reads (length < 50 bp or with a quality value < 20 or having N bases) were removed by fastp [20] (https://github.com/OpenGene/fastp, version 0.20.0). Reads were aligned to the human genome by BWA [21] (http://bio-bwa.sourceforge.net, version 0.7.17), and any hits associated with the reads and their mated reads were removed.

The quality-filtered data were assembled using MEGAHIT [22] (https://github.com/voutcn/megahit, version 1.1.2). Contigs with a length ≥ 300 bp were selected as the final assembling result. Open reading frames (ORFs) from each assembled contigs were predicted using Prodigal [23] (https://github.com/hyattpd/Prodigal, version 2.6.3), and a length ≥ 100 bp ORFs were retrieved.

A non-redundant gene catalogue was constructed using CD-HIT [24] (http://weizhongli-lab.org/cd-hit/, version 4.7) with 90% sequence identity and 90% coverage. The gene abundance for a certain sample was estimated via SOAPaligner [25] (https://github.com/ShujiaHuang/SOAPaligner, version soap2.21 release) with 95% identity.

The best-hit taxonomy of non-redundant genes was obtained by aligning them against the NCBI NR database via DIAMOND [26] (http://ab.inf.uni-tuebingen.de/software/diamond/, version 2.0.13), with an e-value cutoff of 1e-5. Similarly, the functional annotation (KEGG) of non-redundant genes was obtained. Based on the taxonomic and functional annotation and the abundance profile of non-redundant genes, the differential analysis was carried out at each taxonomic, functional, or gene-wise level by the Kruskal‒Wallis test.

### Statistical analysis

Demographic characteristics of participants from the CA and UA were compared. Continuous variables (age, BMI) are presented as the mean ± standard deviation and were compared using independent two-sample t-tests. Categorical variables (sex, smoking status, alcohol consumption and occupation) are presented as counts (percentages) and were compared using chi-square test. To assess the level of HMs contamination in the soil samples, we used the contamination factor (CF). This ratio is obtained by dividing the concentration of each metal in the soil by the background value [27]. For an overall assessment of the extent of soil contamination, the pollution load index (PLI) is used [28]. The calculation formula is as follows:

$$\mathrm{CF}=C_{\mathrm{heavymetal}}/C_{\mathrm{background}}$$

$${\mathrm{PLI}=({\mathrm{CF}}_{1}\times {\mathrm{CF}}_{2}\times \ldots \ldots \;\times {\mathrm{CF}}_{n})}^{1/n}$$

The concentration of HMs in the soil is represented by *C*_heavymetal_, while the background value of HMs is represented by *C*_background_. The level of contamination is categorised according to the size of the CF value as low contamination (CF < 1), moderate contamination (1 < CF < 3), considerable contamination (3 < CF < 6), or very high contamination (CF > 6). PLI > 1 indicates HMs contamination, with larger values indicating more severe contamination. In contrast, PLI < 1 indicates no HMs contamination. We tested soil HMs content between CA and UA using a two-tailed Wilcoxon rank sum test.

All statistical analyses of the microbial data were performed in R (version 4.3.2). Alpha diversity was assessed using the Ace, Chao1 and Sobs indices, which were calculated with the ‘vegan’ and ‘picante’ packages. Differences in each alpha diversity index between the two groups were tested with the Wilcoxon rank-sum test (*P* < 0.05 was considered significant). Beta diversity analysis was performed using principal coordinate analysis (PCoA) based on the Binary-Jaccard distance algorithm. The statistical significance of differences in microbial community structure between groups was assessed using the analysis of similarities (ANOSIM) test with 9999 permutations. The R-value, indicating the proportion of variance explained by the grouping factor, is reported alongside the *P*-value. The SparCC algorithm was used to construct an oral bacterial co-occurrence network based on species-level relative abundance data. The correlation matrix was generated with 100 iterations of bootstrapping to ensure robustness. To objectively determine the adjacency threshold, we applied random matrix theory (RMT), and the adjacency threshold for constructing the adjacency matrix was systematically determined by evaluating the transition in the nearest-neighbour spacing distribution of the correlation matrix eigenvalues. The optimal threshold was selected at the point where this distribution best approximated the Gaussian orthogonal ensemble (GOE) prediction. Following this RMT-based filtering, the resulting correlation matrix was then subjected to statistical filtering: an edge was retained only if the corresponding correlation had an absolute SparCC value > 0.3 and was statistically significant after Benjamini–Hochberg false discovery rate (FDR) correction (adjusted *P* < 0.05). To evaluate whether the observed network topology differed from random, key topological parameters (including the average clustering coefficient, average path distance, modularity, etc.) were calculated for the empirical network. These values were compared descriptively against the distributions (mean ± SD) of the same parameters obtained from 1,000 Erdős–Rényi random networks of identical size and density, which serve as a null model reference. Visualisations of networks were performed using Gephi (version 0.10.0). The functional annotation was based on the Kyoto Encyclopaedia of Genes and Genomes (KEGG) database. The differential abundance of each KEGG pathway between the CA and UA groups was tested using the Wilcoxon rank-sum test. The resulting *P*-values were adjusted for multiple testing using the Benjamini–Hochberg false discovery rate (FDR) correction. A corrected *P*-value (FDR q-value) of less than 0.05 was considered statistically significant.

## Results

### Characteristics of the study participants

This study included 308 participants (CA: *n* = 183; UA: *n* = 125). No statistically significant differences were observed between the two groups in terms of age, BMI, sex distribution, prevalence of current smoking or regular alcohol use, and occupational distribution (all *P* > 0.05; Supplementary Table 1). To definitively control for the strong confounding effects of smoking and alcohol on the oral microbiome, a strictly matched sub-cohort of never smokers and never drinkers was established for subsequent analysis. This sub-cohort comprised 37 eligible participants (CA: *n* = 26; UA: *n* = 11). Within this sub-cohort, the two groups remained comparable in terms of age, BMI, sex, and occupation (all *P* > 0.05; Supplementary Table 2), thus isolating the comparisons from these key lifestyle confounders.

### Analysis of HMs contamination of soil

**Figure 2.**
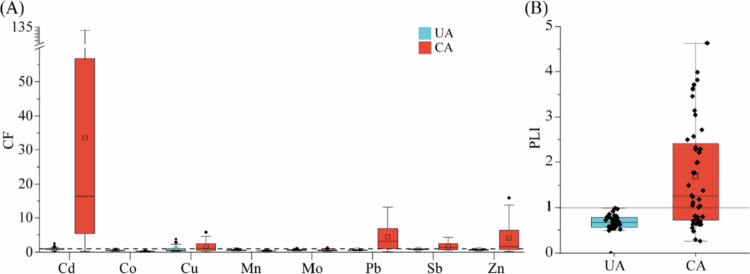
Assessment of soil heavy metal pollution levels. The contamination status of soils from the uncontaminated area (UA, blue) and the contaminated area (CA, red) is quantified using two standard indices: (A) the contamination factor (CF) and (B) the pollution load index (PLI) for eight heavy metals (As, Cd, Cr, Cu, Hg, Ni, Pb and Zn). The dashed line in each panel indicates the threshold (CF = 1; PLI = 1) between the uncontaminated and contaminated levels. The results demonstrated significantly elevated CF and PLI values in the CA group, confirming the intended severe pollution gradient between the two study sites.

Long-term non-ferrous metal mining and smelting activities in the study area resulted in local HMs above the limits. We analysed the concentrations of Mo, Sb, Pb, Mn, Co, Cu, Zn and Cd in agricultural soils. *n* the UA, the levels of all HMs were below the local soil background values and were significantly lower than those in the CA (*P* < 0.05, Table 1). In CA, the concentrations of Sb, Pb, Cu, Zn and Cd exceeded their respective background values, which is consistent with mining and smelting activities being the primary anthropogenic source of soil contamination. CF and PLI were used to assess the level of HMs pollution and the degree of soil contamination in all the samples (Figure 2). In the UA, both CF and PLI values were below 1, indicating low contamination, with data points showing relatively centralised distributions. Conversely, in the CA, the CF for Cd was 16.36, indicating very high contamination and identifying Cd as the most severe pollutant, followed by Pb (CF = 3.10, considerable contamination).

This indicated that Cd and Pb were the main pollutants in the CA. The CF of Zn, Sb and Cu were 1.62, 1.40 and 1.14, respectively, which represented moderate contamination. The CF of Co, Mn and Mo were lower than 1, indicating low contamination (Figure 2A). The PLI for the CA was 1.26, indicating mild overall pollution (Figure 2B). Notably, the dispersion of CF and PLI values for Cd in the CA was substantially greater than in the UA, reflecting the high variability and extreme spatial heterogeneity of HM contamination. Spearman's correlation was used to evaluate the correlation of HMs between CA and UA (Supplementary Figure 1). In the UA soils, no significant correlation was found between Cd and the other HMs (*P* > 0.05). In contrast, in the CA soils, all HMs showed significant positive correlations with each other (*P* < 0.001). These findings further demonstrates that their common anthropogenic source is mining and smelting activities.

### Biomarkers response

**Figure 3.**
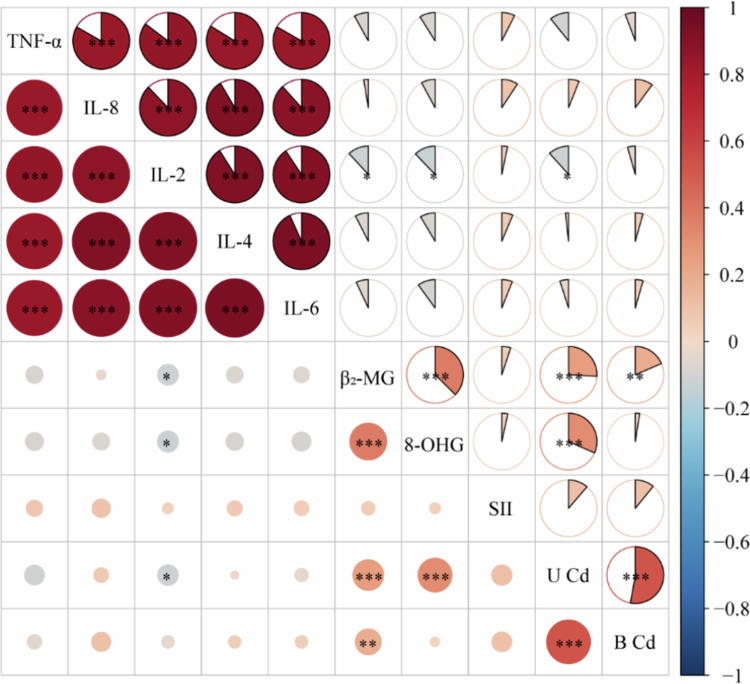
Correlation analysis between urinary and blood biomarkers. The heatmap displays the matrix of pairwise Pearson correlation coefficients (*r*) among all measured biomarkers (U, urinary; B, blood) across all study participants. Each circle represents the correlation between two indicators. The direction and strength of the correlation are jointly encoded: the colour indicates the direction (red, positive; blue, negative), and the size of the circle is proportional to the absolute value of the correlation coefficient |*r*|. Statistical significance levels (two-tailed test) are indicated by asterisks within the circles. (***P < 0.001, **P < 0.01, *P < 0.05).

Blood Cd and urinary Cd are recognised biomarkers of internal exposure, reflecting long-term and recent Cd exposure, respectively. Consistent with the soil contamination findings, participants in the CA group presented significantly elevated Cd exposure levels, as evidenced by higher concentrations of both blood Cd and urinary Cd compared to the UA group (*P* < 0.001, Table 2). More importantly, these exposures were associated with early signs of adverse health effects, including impaired renal function (elevated urinary β_2_-MG and 8-OHG) and altered immune markers (decreased plasma IL-2) (*P* < 0.05, Table 2). To further explore these associations, a subset of 297 participants with paired blood and urine samples was analysed. Linear correlation analysis revealed significant positive correlations between the internal exposure markers (blood Cd, urinary Cd) and the renal effect markers (*P* < 0.01, Figure 3). This correlation analysis reinforces the observed link between Cd exposure and markers of renal stress.

**Table 2.** Urinary and blood indicators of participants in the uncontaminated (UA) and contaminated (CA) areas.

Indicators	Groups	M (Q1, Q3)	*P*-value
Urine Cd	UA (*n* = 117)	0.780 (0.544, 1.024)	<0.001
(µg/L)	CA (*n* = 180)	5.151 (3.182, 7.330)	
Blood Cd	UA (*n* = 125)	0.080 (0.080, 0.478)	<0.001
(µg/L)	CA (*n* = 183)	3.995 (1.827, 6.675)	
Urine β_2_-MG	UA (*n* = 117)	247.791 (174.707, 345.979)	0.042
(µg/g Cr)	CA (*n* = 180)	283.525 (184.624, 497.046)	
Urine 8-OHG	UA (*n* = 117)	246.320 (169.824, 356.343)	0.001
(ng/g Cr)	CA (*n* = 180)	294.143 (192.555, 467.216)	
SII (1000 cell/µL)	UA (*n* = 125)	384.632 (253.847, 513.656)	0.039
	CA (*n* = 183)	421.200 (310.471, 584.250)	
Plasma IL-2	UA (*n* = 125)	230.089 (203.261, 272.747)	0.028
(pg/ml)	CA (*n* = 183)	199.124 (166.942, 246.456)	
Plasma IL-4	UA (*n* = 125)	613.800 (499.800, 742.500)	0.701
(ng/L)	CA (*n* = 183)	609.514 (486.935, 734.583)	
Plasma IL-6	UA (*n* = 125)	26.203 (20.629, 30.648)	0.295
(pg/ml)	CA (*n* = 183)	23.597 (19.604, 29.612)	
Plasma IL-8	UA (*n* = 125)	1262.143 (990.000, 1612.143)	0.267
(ng/L)	CA (*n* = 183)	1536.000 (1183.993, 1888.500)	
Plasma TNF-α	UA (*n* = 125)	153.378 (141.560, 184.579)	0.059
(ng/L)	CA (*n* = 183)	154.359 (129.902, 175.522)	

Note: The data are presented as median and interquartile range (IQR) with the sample size in brackets. Differences between the UA and CA groups for each biomarker were assessed using the Mann‒Whitney U test. *P* < 0.05 was considered statistically different.

### Analysis of the structure and diversity of oral bacterial communities

**Figure 4.**
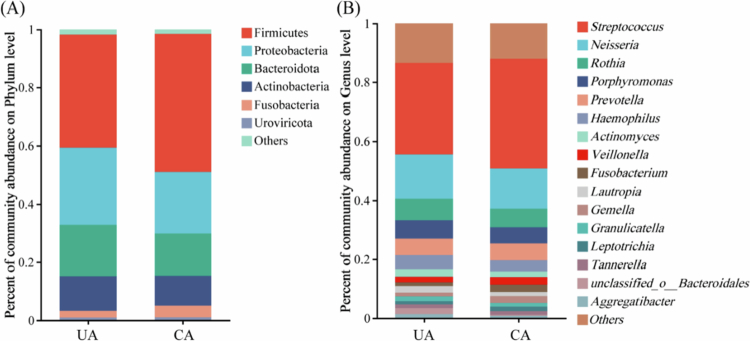
Bacterial community composition of the buccal mucosa in the uncontaminated and contaminated areas. Stacked bar charts compare the mean relative abundance of bacterial communities between the uncontaminated area (UA) and the contaminated area (CA) at (A) the phylum level and (B) the genus level. Only the top 16 most abundant taxa are displayed, with all others grouped into *Others*. The colours represent different bacterial taxa.

**Figure 5.**
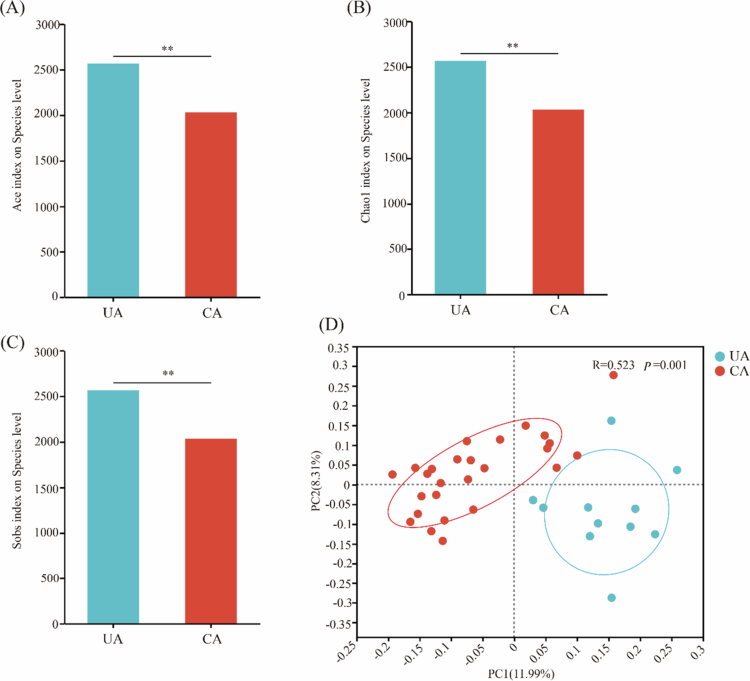
Analysis of bacterial community diversity in the oral buccal mucosa. Diversity was assessed for the uncontaminated (UA, blue) and contaminated (CA, red) areas. (A–C) Alpha diversity: Bar charts (mean ± SD) showing the (A) ACE, (B) Chao1 and (C) Sobs indices. Between-group differences were evaluated using the Wilcoxon rank-sum test (*P* < 0.01). (D) Beta diversity: principal coordinate analysis (PCoA) plot based on Binary-Jaccard distances. Each point represents an individual sample. The statistical significance of the community structure separation was tested with ANOSIM (R = 0.523, *P* = 0.001).

UA and CA identified a total of 6 phyla and 16 genera with relative abundances above 0.01%. Firmicutes, Proteobacteria, Bacteroidota and Actinobacteria were the four dominant phyla in both groups (Figure 4A). At the genus level, *Streptococcus*, *Neisseria*, *Rothia, Porphyromonas* and *Prevotella* were the five dominant phyla in both groups (Figure 4B). Notably, the overall composition of the oral bacterial communities differed between the two areas. Macro-genome sequencing of the 37 buccal mucosa samples yielded a total of 1,048,549,410 bp. After de-redundancy, 1,357,440 bp were retained for downstream analysis. The alpha diversity indices (Ace, Chao1 and Sobs) were significantly higher in the UA than in the CA (*P* < 0.01, Figure 5A‒C). Beta diversity, analysed by PCoA based on Binary-Jaccard distance, revealed a significant difference in bacterial community structure between the UA and CA groups (R = 0.523, *P* = 0.001, Figure 5D). The first and second principal components together explained 20.30% of the overall variation. These findings demonstrate distinct oral microbiome profiles associated with the two exposure environments.

### Co-occurrence network analysis of bacterial communities in the oral buccal mucosa

**Figure 6.**
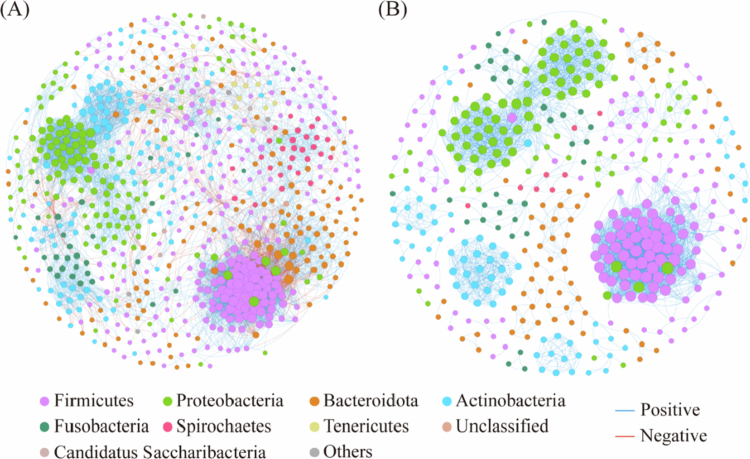
Construction of an oral mucosal bacterial co-occurrence network based on species level. Bacterial association networks were constructed separately for the (A) uncontaminated (UA) and (B) contaminated (CA) areas using the SparCC algorithm. Only robust (SparCC |ρ| > 0.3) and statistically significant correlations (FDR-adjusted *P* < 0.05) are shown. In each network, node size scales with the number of connections (degree), and node colour represents the bacterial phylum. Edges (lines) represent significant pairwise associations (red, positive; blue, negative). The comparative visualisation reveals distinct topological structures, with the CA network exhibiting reduced complexity and altered modularity compared to the UA network.

**Figure 7.**
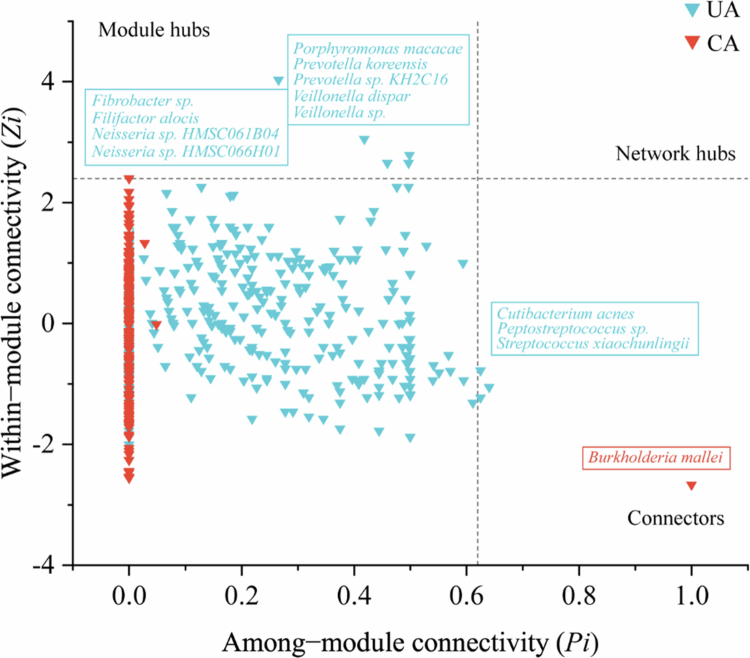
Topological role of the bacterial co-occurrence network in the buccal mucosa. The scatter plot classifies each species into a topological role by plotting its within-module connectivity (*Zi*) against its among-module connectivity (*Pie*) for the uncontaminated (UA, blue triangles) and contaminated (CA, red triangles) areas. Theoretical thresholds (dashed lines at *Zi* = 2.5 and *Pie* = 0.62) define four roles: Peripherals (*Zi* < 2.5, *Pie* < 0.62), Connectors (*Zi* < 2.5, *Pie* > 0.62), Module hubs (*Zi* > 2.5, *Pie* < 0.62), and Network hubs (*Zi* > 2.5, *Pie* > 0.62). Species with connector or hub roles are considered keystone taxa.

To explore the relationship between the oral microbiome and health, species-level bacterial co-occurrence networks for the buccal mucosa were constructed using the SparCC algorithm. The degree distributions of both the UA and CA networks conformed to power law distributions (R^2^ = 0.838 and R^2^ = 0.940), indicating scale-free networks. Structurally, the network of UA yielded 749 nodes and 7,285 edges, while the network of the CA contained 427 nodes and 3,372 edges (Table 3). Consistent with this reduced size, the CA network exhibited higher average clustering coefficient (avgCC), density (D) and modularity (M), but a lower average degree (avgK) and average path distance (GD) compared to the UA network. Consistent with this reduced size, the CA network exhibited higher average clustering coefficient (avgCC), density (D), and modularity (M), but a lower average degree (avgK) and average path distance (GD) compared to the UA network (Table 3). Simulations of random node removal indicated that the CA network was more susceptible to disruption (Supplementary Figure 2). These structural differences suggest an altered pattern of bacterial interactions in the CA group. The network nodes of UA and CA were dominated by Firmicutes (37.52 and 35.60%), Proteobacteria (20.03 and 23.89%), Bacteroidota (17.49 and 15.22%), and Actinobacteria (14.15 and 15.46%, Figure 6), which together accounted for 89.19 and 90.17% of all nodes. The observed decrease in network complexity and increase in modularity in the CA may represent a community-level response correlated with HMs contamination. The topological roles of nodes in the network were categorised based on the thresholds of within-module connectivity (*Zi*) and among-module connectivity (*Pie*). No network hubs were identified in either group. In the UA network, 12 keystone taxa were identified (Figure 7): 3 connectors (*Cutibacterium acnes*, *Peptostreptococcus sp*., and *Streptococcus xiaochunlingii*) and 9 module hubs (*Fibrobacter sp*., *Filifactor alocis*, *Neisseria sp*. *HMSC061B04*, *Neisseria sp*. *HMSC066H01*, *Porphyromonas macacae, Prevotella koreensis*, *Prevotella sp*. *KH2C16*, V*eillonella dispar*, and *Veillonella sp*.). These keystone taxa belong to the phyla Firmicutes, Bacteroidota, Proteobacteria, Actinobacteria and Fibrobacterota (Supplementary Table 3). In contrast, only one keystone taxon, classified as a connector (*Burkholderia mallei*, phylum Proteobacteria), was detected in the CA network (Figure 7). Taken together, the CA network was characterised by reduced complexity, increased modularity, and a marked depletion of keystone taxa.

**Table 3.** Topological properties of bacterial co-occurrence and randomised networks in the buccal mucosa of the uncontaminated (UA) and contaminated (CA) areas.

	UA		CA	
	Empirical	Random	Empirical	Random
Total nodes	749	NA	427	NA
Total links	7285	NA	3372	NA
Positive edges	86.33%	NA	100.00%	NA
Negative edges	13.67%	NA	0.00%	NA
R square of power-law	0.838	NA	0.940	NA
Average degree (avgK)	19.453	NA	15.794	NA
Average clustering coefficient (avgCC)	0.537	0.019 ± 0.003	0.643	0.023 ± 0.005
Average path distance (GD)	4.843	2.695 ± 0.012	2.084	2.727 ± 0.017
Geodesic efficiency (E)	0.254	0.402 ± 0.001	0.636	0.404 ± 0.002
Harmonic geodesic distance (HD)	3.931	2.488 ± 0.007	1.572	2.475 ± 0.011
Centralisation of degree (CD)	0.102	0.102 ± 0.000	0.127	0.127 ± 0.000
Centralisation of betweenness (CB)	0.086	0.023 ± 0.002	0.004	0.033 ± 0.005
Centralisation of stress centrality (CS)	64.903	0.139 ± 0.014	0.088	0.132 ± 0.019
Centralisation of eigenvector centrality (CE)	0.905	0.816 ± 0.005	0.878	0.79 ± 0.006
Centralisation of closeness centrality (CCL)	0.004	0.17 ± 0.081	0.001	0.039 ± 0.046
Density (D)	0.026	0.026 ± 0.000	0.037	0.037 ± 0.000
Modularity (M)	0.572	0.165 ± 0.003	0.647	0.173 ± 0.004
Transitivity (Trans)	0.635	0.14 ± 0.002	0.796	0.216 ± 0.004
Connectedness (Con)	0.924	0.998 ± 0.003	0.096	0.972 ± 0.015
Efficiency	0.973	0.988 ± 0.000	0.627	0.983 ± 0.000
Hierarchy	0	0.026 ± 0.000	0	0.037 ± 0.000
Lubness	1	0.255 ± 0.009	1	0.368 ± 0.013

Note: For the empirical networks, key topological properties are presented as mean ± standard deviation (SD) from the network calculation. For the randomised networks, values are presented as mean ± SD derived from 1,000 randomisations. NA indicates that no data were available in the randomised algorithm.

### Functional prediction of bacterial communities in the oral buccal mucosa

**Figure 8.**
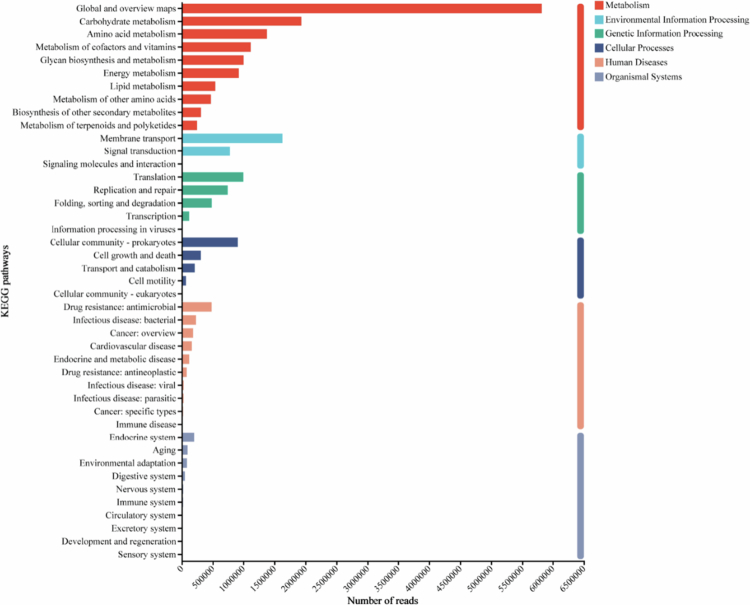
Pathway classification statistics of KEGG functional annotation in oral buccal mucosa bacteria. The bar chart displays the total gene abundance mapped to KEGG pathways. Pathways are organised by Level 1 categories (colour-coded: metabolism, genetic information processing, environmental information processing, cellular processes, human diseases and organismal systems) and further detailed by Level 2 subcategories (y-axis). For clarity, only the top 10 most abundant Level 2 pathways within each Level 1 category are displayed. The x-axis shows the gene abundance (in reads).

**Figure 9.**
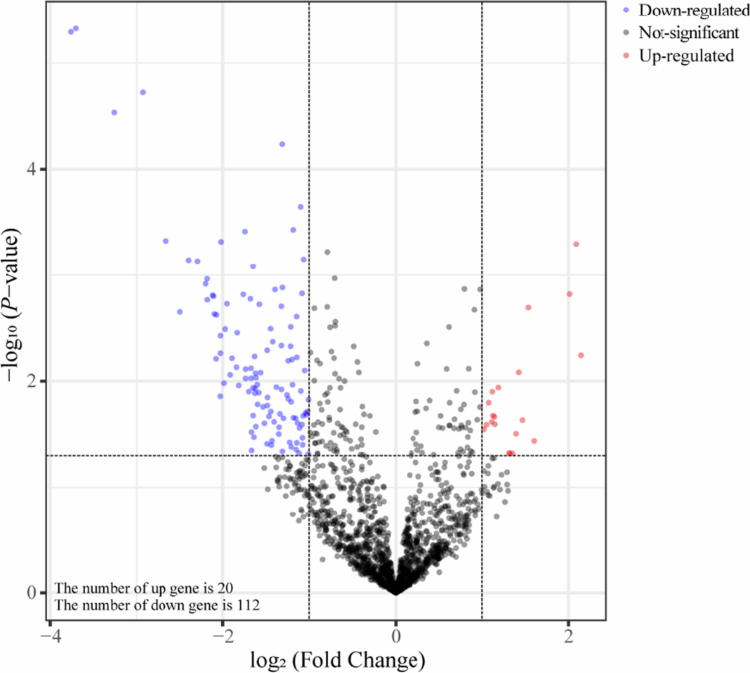
Differential abundance analysis of KEGG metabolic pathway genes in oral buccal mucosa bacteria. Volcano plot displaying KEGG orthologs (KOs) that are differentially abundant between the contaminated (CA) and uncontaminated (UA) areas. Each point represents a functional gene (KO). The log₂-fold change (CA/UA) is plotted on the x‑axis against the statistical significance (‑log₁₀(*P*‑value)) on the y‑axis. Significantly differentially abundant genes (coloured points) were defined by an absolute log₂-fold change ≥ 1 and a false discovery rate (FDR)-adjusted *P*‑value < 0.05, with red indicating upregulation and blue indicating downregulation in the CA group relative to the UA group. The grey points represent non‑significant genes.

Functional annotation was performed using the KEGG database and tested functional differences in metabolic pathways and human disease between the two groups. Analysis identified 6 KEGG level 1 pathways (Figure 8). The most abundant functional category was metabolism, the pathways of which were selected for further analysis. The prediction based on the KEGG level 2 pathways showed that the two groups exhibited significant functional differences (*P* < 0.05, Figure 9). Differential gene analysis revealed 20 genes with significantly increased abundance and 112 genes with significantly decreased abundance in the CA compared to the UA (Supplementary Table 4). The three genes whose expression increased the most were *gumG*, *asrC* and *E3.5.4.16*. These genes are associated with exopolysaccharide biosynthesis (ko0054), sulphur metabolism (ko00920), microbial metabolism in diverse environments (ko01120), folate biosynthesis (ko00790) and the biosynthesis of cofactors (ko01240). Conversely, the three genes whose expression decreased the most were *POP2*, *fadN* and *DCAA*, which are implicated in pathways including butanoate metabolism (ko00650), alanine, aspartate, and glutamate metabolism (ko00250), benzoate degradation (ko00362), fatty acid metabolism (ko01212), carbon metabolism (ko01200), fatty acid degradation (ko00071) and caprolactam degradation (ko00930). Analysis of specific level 2 pathways within the metabolism and human diseases categories revealed distinct patterns (Supplementary Figure 3). Within metabolism, D-amino metabolism (ko00470) was significantly more abundant in the CA, whereas propanoate metabolism (ko00640) was significantly lower (*P* < 0.05; Supplementary Figure 3A). Within human diseases, pathways for central carbon metabolism in cancer (ko05230) and microRNAs in cancer (ko05206) were significantly enriched in the CA, while pathways for pertussis (ko05133) and legionellosis (ko05134) were more abundant in the UA (*P* < 0.05; Supplementary Figure 3B). At KEGG level 3, most pathways under metabolism and human diseases were significantly enriched in the CA (Supplementary Figure 4). Among the metabolic pathways, the top three pathways significantly enriched in the CA were valine, leucine and isoleucine degradation (ko00280) and oxidative phosphorylation (ko00190); the top three pathways enriched in the UA were teichoic acid biosynthesis (ko00552), acarbose and validamycin biosynthesis (ko00525), and peptidoglycan biosynthesis (ko00550) (Supplementary Figure 4A). Among the human disease pathways (Supplementary Figure 4B), the top three pathways significantly enriched in the CA were non-alcoholic fatty liver disease (ko04932) and spinocerebellar ataxia (ko05017); the top three pathways in the UA were *Staphylococcus aureus* infection (ko05150) and bacterial invasion of epithelial cells (ko05100).

### Correlation of oral bacteria with biomarkers

**Figure 10.**
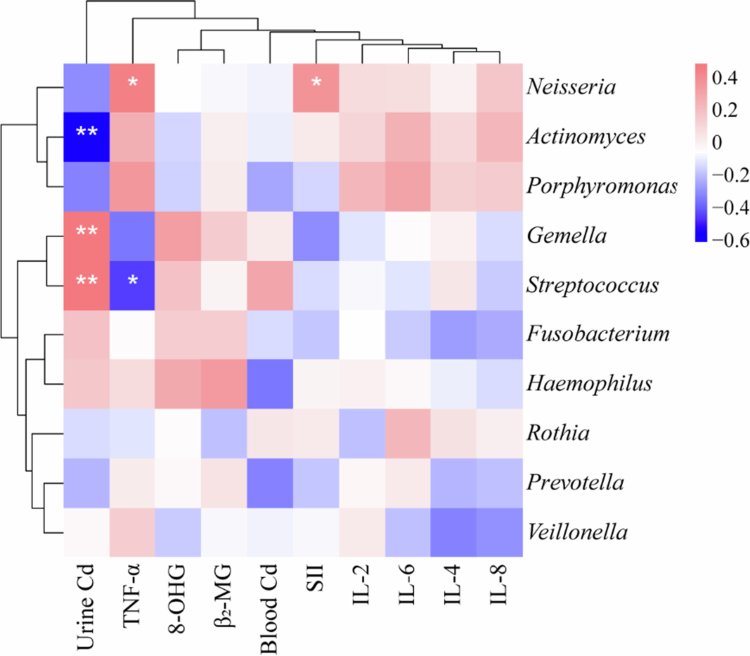
Correlation analysis between oral bacterial taxa and biomarkers. The heatmap depicts the pairwise correlation coefficients between oral bacterial taxa (rows) and measured biomarkers (columns). The colour intensity and hue indicate the direction and strength of the correlation: red represents positive correlations, blue represents negative correlations, and white indicates no significant correlation. The strength of the correlation is proportional to the colour saturation. Statistically significant correlations are denoted by asterisks within the cells: (**0.001 < *P* < 0.01, *P < 0.05).

Significant correlations were observed between the relative abundance of specific oral bacterial genera and systemic biomarkers of HMs exposure (Figure 10). Notably, the abundance of Actinomyces exhibited a negative correlation with urinary Cd levels. In contrast, the abundances of Gemella and Streptococcus were positively correlated with urinary Cd (Figure 10, Supplementary Table 5). These genus-specific shifts correlated not only with an exposure biomarker (urinary Cd) but also with an early-effect biomarker (TNF-*α*). However, the lack of correlation with blood Cd highlights a potentially different kinetic profile, meriting further investigation. However, no significant correlations were detected between these bacterial genera and blood Cd levels, suggesting a distinct kinetic relationship that warrants further study.

## Discussion

The harmful effects of long-term HMs exposure have been reported in many studies in various aspects [29,30], and the susceptibility of oral microorganisms have also been reported due to their susceptibility to environmental factors [31]. However, few studies have combined biomarkers and oral microorganisms to analyse the effects of long-term HMs exposure. This study evaluated the health threats of long-term HMs exposure from both perspectives. A key challenge in such observational studies is distinguishing specific exposure-related alterations from background variations. Therefore, we employed a comparative study design, sampling participants from a well-characterised, heavily contaminated area (CA) and a matched uncontaminated area (UA). We posited that any microbiome alterations associated with HMs exposure would be most pronounced and thus more readily detectable in a highly exposed population. Our integrated analysis revealed that long-term environmental HMs exposure was associated with early signs of renal impairment and immune modulation. Concomitantly, we observed distinct alterations in the oral microbiome of the CA participants, including reduced alpha diversity, decreased network stability, a shift in keystone taxa, and significant functional reprogramming. Furthermore, specific oral bacterial genera showed significant correlations with internal exposure and effect biomarkers. These findings collectively suggest that the oral microbial profile may reflect the host exposure status and biological response. This comparative approach provides critical preliminary evidence on exposure-related microbial signatures, which can inform future mechanistic and longitudinal validation studies.

First, we assessed the HMs contamination in the study areas. The soils in the CA were primarily polluted by Cd and Pb, with their concentrations showing notable spatial variability. While such variability can arise from the weathering of natural parent rocks [32], a high degree often points to anthropogenic sources [33]. In our data, all the detected HMs in the CA showed strong and significant positive correlations with each other. These correlations were substantially higher than those found in the UA, which strongly suggests a common, human-derived origin for these pollutants [34]. This inference is further supported by the documented long-term practice of wastewater irrigation in the CA [35]. Collectively, this evidence leads us to infer that non-ferrous metal smelting and mining activities likely polluted the local water and soil, with subsequent wastewater irrigation further exacerbating the contamination levels.

Next, we evaluated the potential health effects associated with long-term HMs exposure using biomarkers. The kidney is a primary organ for Cd accumulation, and long-term exposure is linked to tubular and glomerular damage and may lead to irreversible kidney disease [36]. In this study, urinary β_2_-MG and 8-OHG levels were significantly higher in the CA. β_2_-MG is a sensitive biomarker of proximal renal tubular dysfunction [37]. Cd-induced damage to renal tubules at urinary β_2_-MG levels above 300 µg/g increases the risk of death from kidney disease in the population [37]. Thus, our data indicate potential renal tubular injury in the CA group, with levels currently below the high-risk threshold. Urinary 8-OHG is a biomarker of the biological effects of oxidative DNA damage and can indicate the progression of disease complications such as diabetes, cancer and some bacterial infections [38]. Its elevation suggests that Cd exposure was associated with oxidative DNA damage, which poses a potential risk to human health. Moreover, the plasma levels of IL-2 in the CA were significantly lower than those in the UA, whereas the SII values were significantly higher than those in the UA. IL-2 is a critical cytokine for T cell peripheral tolerance and immunity and is an important target for bioengineering [39]. The SII is based on a combination of lymphocyte, neutrophil, and platelet counts that reflect the body's systemic immune status [11]. As such, we proposed that environmental Cd exposure may contribute to altered immune status, which could indirectly increase the risk of disease in humans. Previous studies have confirmed that urinary β_2_-MG was significantly and positively correlated with blood Cd and urinary Cd, which is consistent with the findings of this study [40]. This finding further supports the association between Cd exposure and kidney damage, where the extent of damage is positively correlated with the level of exposure [40]. Furthermore, there was a significant positive correlation between urinary 8-OHG and urinary Cd, which is consistent with the notion that Cd exposure can cause oxidative damage. Additionally, the strong correlations among pro-inflammatory cytokines likely reflect their coordinated roles in the immune system.

Third, to assess the health impacts of long-term HMs exposure from a novel perspective, we analysed changes in the oral microbiome. The humid environment of the oral cavity provides excellent conditions for the growth of various microorganisms [41]. Our analysis revealed distinct differences in the diversity and composition between the UA and CA bacterial communities. The dominant phyla in both groups were Firmicutes, Proteobacteria, Bacteroidota, and Actinobacteriota, with *Streptococcus*, *Neisseria*, *Rothia* and *Porphyromonas* being the dominant genera. This profile is generally consistent with previous reports and indicated that the population selected for this study was representative [42,43]. *Streptococcus* and *Neisseria* had the highest relative abundances. *Streptococcus* is the dominant bacterium in the oral cavity [44], and *Neisseria* are recognised as core genera by the global core bacterial microbiome [42]. Importantly, we found a significant reduction in bacterial community diversity in the CA. This finding aligns with prior studies indicating that HMs exposure can alter oral microbial ecology [45] and may reflect a state of microbial dysbiosis associated with environmental stress.

Microbial co-occurrence network analysis can be used to determine microbial interactions and identify keystone taxa [46,47]. In both groups, associations between bacterial taxa were predominantly positive, a common pattern in microbial networks often linked to cooperative or co-occurrence relationships [48]. Notably, the CA network contained a higher proportion of positive correlations than did the UA network, aligning with reports that microbial communities under stress may exhibit increased cooperative signatures [49,50]. Furthermore, although the UA network demonstrated greater overall complexity, the CA network was characterised by higher modularity and stronger local clustering. These distinct network properties suggest a community-wide structural shift associated with long-term HMs exposure. One possible interpretation is that increased symbiotic relationships and enhanced local connectivity represent a strategic response of the oral microbiota to chronic stress. Recognising modules helps identify species with similar ecological roles or metabolic pathways [51]. Here, the distribution of Firmicutes, Proteobacteria, and Actinobacteria formed distinct modules, whereas Bacteroidota showed little aggregation, especially in the CA. Taxa with fewer and weaker network connections may be more susceptible to community restructuring during environmental perturbation, which could explain the lower relative abundance of Bacteroidota observed in the CA. Keystone taxa are critical in shaping the structure and functioning of biomes and play an important role in co-occurrence networks [52]. We identified 12 keystone taxa in the UA network. Many are recognised as beneficial or core members of a healthy oral microbiome. For instance, *Veillonella sp*. is an early settler in the oral cavity [53]. The genus *Veillonella* is one of the main anaerobic bacteria that is beneficial in the oral cavity. The genera *Prevotella* and *Actinomyces* have a broad interspecific association and play a core role in establishing and maintaining the complexity of biofilms [54]. *Streptococcus xiaochunlingii* is a core component of the healthy human oral cavity ecosystem [55]. *Neisseria sp*. *HMSC061B04* and *Neisseria* sp. *HMSC066H01* belongs to the genus *Neisseria*. *Neisseria* has the ability to inhibit the proliferation of oral squamous cell carcinoma (OSCC) cells [56]. *Porphyromonas macacae* is one of the top ten anaerobic flora in the oral cavity of healthy humans [57]. The other four keystone taxa are mainly from other parts of the human body. For example, the skin, vagina and other parts of the body are affected. The role of this species in the oral cavity remains to be further studied [58]. Only one keystone taxon, *Burkholderia mallei*, was identified in the CA. *Burkholderia mallei* is a parthenogenetic intracellular pathogen that causes human and zoonotic diseases [59]. Taken together, these results indicate a marked shift in keystone taxa. The UA network was dominated by beneficial or commensal core genera, while the CA network’s sole keystone taxon was a pathogen. This restructuring suggests that prolonged HMs exposure may alter the microbial hierarchy, potentially increasing the ecological influence of taxa with pathogenic potential.

KEGG pathway analysis was employed to assess the functional impact of HMs exposure on the oral microbiota, focusing on metabolism and human disease pathways [60]. The results revealed a functional profile in the CA group indicative of microbial adaptation to stress, which may concurrently disrupt host‒microbe symbiosis. The most salient adaptive responses were the significant enrichment of exopolysaccharide (EPS) biosynthesis and sulphur metabolism pathways in the CA group. The upregulation of EPS-related genes suggests an enhanced capacity for biofilm formation. Critically, microbial EPS are known not only to form a protective barrier but also to directly chelate HMs ions, reducing their bioavailability and toxicity [61–63]. Thus, we posit that increased EPS synthesis represents a primary microbial strategy for HMs detoxification and survival. Concurrently, enhanced sulphur metabolism, potentially involving genes related to glutathione synthesis, is likely another key detoxification mechanism. Sulphur (S) is integral to synthesising antioxidants such as glutathione, which is crucial for mitigating HMs-induced oxidative stress [64–66]. Notably, the co-enrichment of these two pathways suggests a non-redundant, multi-layered defence strategy: while EPS provides a first line of extracellular sequestration, enhanced sulphur metabolism supports intracellular detoxification and oxidative stress management. This functional synergy indicates robust and coordinated microbial adaptation to HMs stress. Together, the upregulation of EPS and sulphur metabolism pathways constitutes a coherent microbial defence system against HMs toxicity. Conversely, we observed a concurrent downregulation of genes involved in several nutrient metabolism pathways, including amino acid, fatty acid, and short-chain fatty acid (SCFA) production (e.g. butyrate and propionate). This pattern may reflect a metabolic trade-off, where resources are redirected from growth-associated biosynthesis towards essential stress-response functions [67]. Since SCFAs such as butyrate are vital for maintaining mucosal integrity and immune homoeostasis [68,69], their diminished production potential is a plausible mechanistic link between HMs-altered microbiota and adverse local health outcomes, such as compromised epithelial barrier function or dysregulated immune responses. The enrichment of D-amino acid metabolism alongside altered fatty acid profiles may further indicate broader microbial structural adaptations to stress [70]. The enrichment of human disease pathways, such as central carbon metabolism in cancer and miRNA pathways in cancer, requires cautious interpretation. These KEGG annotations primarily arise from the homology of conserved bacterial metabolic genes with those annotated in human disease pathways, rather than signifying direct oncogenic processes in the host [71,72]. A more conservative and appropriate interpretation is that HMs exposure induces broad dysregulation of core microbial metabolism, the functional consequences of which are catalogued under these disease-associated categories in the database. Similarly, the decreased abundance of ‘pertussis’ and ‘legionellosis’ pathways, which encompass general bacterial metabolic genes, likely reflects a global shift in microbial metabolic states rather than specific changes in pathogenicity [73].

Finally, we observed significant correlations between exposure and early-effect biomarkers and the relative abundance of specific oral bacterial taxa. These correlations reinforce the association between HMs exposure and a shift in oral microbiota composition. Overall, our findings demonstrate that the oral microbiome undergoes reproducible and functionally consequential changes associated with long-term HMs exposure. The correlation of specific microbial features with clinical biomarkers, combined with the ease of oral sampling, positions the oral microbiome as a promising candidate for a novel, ecosystem-level biomarker of exposure and early biological effect. It is important to emphasise that these interpretations derive from a correlational study design. Therefore, future large-scale longitudinal studies utilising more integrated analytical approaches are essential to validate the specific microbial signatures identified here and to evaluate their diagnostic performance for monitoring HMs exposure and associated health risks.

While this study provides novel insights into the associations between HMs exposure and the oral microbiome, several limitations must be considered. First, the cross-sectional design precludes causal inference; although we observed robust correlations, the temporal sequence and direct causality cannot be established. Future longitudinal studies are required to confirm the directional effects of HMs. Second, the modest sample size may limit the statistical power for complex analyses and generalisability. Nevertheless, the consistent signals across multiple analytical levels strengthen the credibility of our core findings, which should be validated in larger cohorts. Third, functional predictions based on metagenomic data and the KEGG database are inferential. The enrichment of disease-related pathways likely reflects gene homology rather than direct host pathogenesis, highlighting the need for multi-omics validation (e.g. metatranscriptomics, metabolomics) to confirm pathway activity and quantify key metabolites.

## Conclusion

In conclusion, this study demonstrates a significant environmental-to-human continuum of HMs pollution. The soil in the mining area was heavily contaminated with HMs, which was associated with their elevated accumulation in the participants’ bodies. This elevated internal dose was further correlated with biomarkers indicative of potential kidney stress and altered immune profiles. Our findings reveal that long-term HMs exposure is associated with a distinct alterations in the oral microbial community. These HMs-associated microbial shifts, characterised by changes in community structure, function, and network architecture, may contribute to an increased health risk in the exposed population. Specifically, microbial networks under exposure exhibited adaptations such as increased modularity, suggesting a potential microbial strategy to resist HMs stress through changes in species interconnectivity. Moreover, long-term HMs exposure was linked to a restructuring of keystone taxa and a shift in dominant genera, with an increased relative abundance of taxa that include recognised pathogens. This alteration in the microbial ecosystem presents a plausible, microbiota-mediated pathway through which HMs could pose a threat to host health. At the functional level, HMs exposure was significantly correlated with disruptions in multiple bacterial metabolic pathways, potentially undermining microbial contributions to host homoeostasis. The concurrent observation of altered host immunity suggests a possible interplay between HMs-driven dysbiosis and immune dysfunction, which could enhance population vulnerability. Overall, this study establishes compelling correlative links between environmental HMs exposure, oral microbiome dysbiosis, and early host biological responses. These findings highlight the potential of the oral microbiota as a sensitive biomarkers of exposure and early effects. Future longitudinal and mechanistic studies are warranted to confirm causal relationships and elucidate the detailed molecular mechanisms underlying these associations.

## Supplementary Material

Revised Supplementary Materials.docx Revised Supplementary Materials.docx

## Data Availability

Data supporting the findings of this study are available in this article and its Supplementary Information, or from the corresponding author on request. The raw sequence data have been deposited in the NCBI Sequence Read Archive under BioProject accession numbers: https://www.ncbi.nlm.nih.gov/,PRJNA979792.
