# Supplementary material for: Oral microbiome alterations and their association with long-term heavy metal exposure and early health effects
Source: J Oral Microbiol. 2026 Mar 23;18(1):2647511. doi: 10.1080/20002297.2026.2647511 (PMC13011095; doi:10.1080/20002297.2026.2647511)
Supplement: Revised Supplementary Materials.docx [file ZJOM_A_2647511_SM2400.docx]

***Supplementary materials***

**Supplementary Figures**

**Supplementary Figure 1. Correlation analysis of soil heavy metal concentrations.**

The heatmap displays the correlation matrix for (A) heavy metal uncontaminated (UA) and (B) contaminated areas (CA). The color and size of each circle jointly represent the strength and direction of the correlation: color indicates the direction (red: positive, blue: negative, white: weak), and circle size is proportional to the absolute value of the correlation coefficient. Statistical significance is denoted by asterisks within the circles. (****P*<0.001, **0.001<*P*<0.01, **P*<0.05).


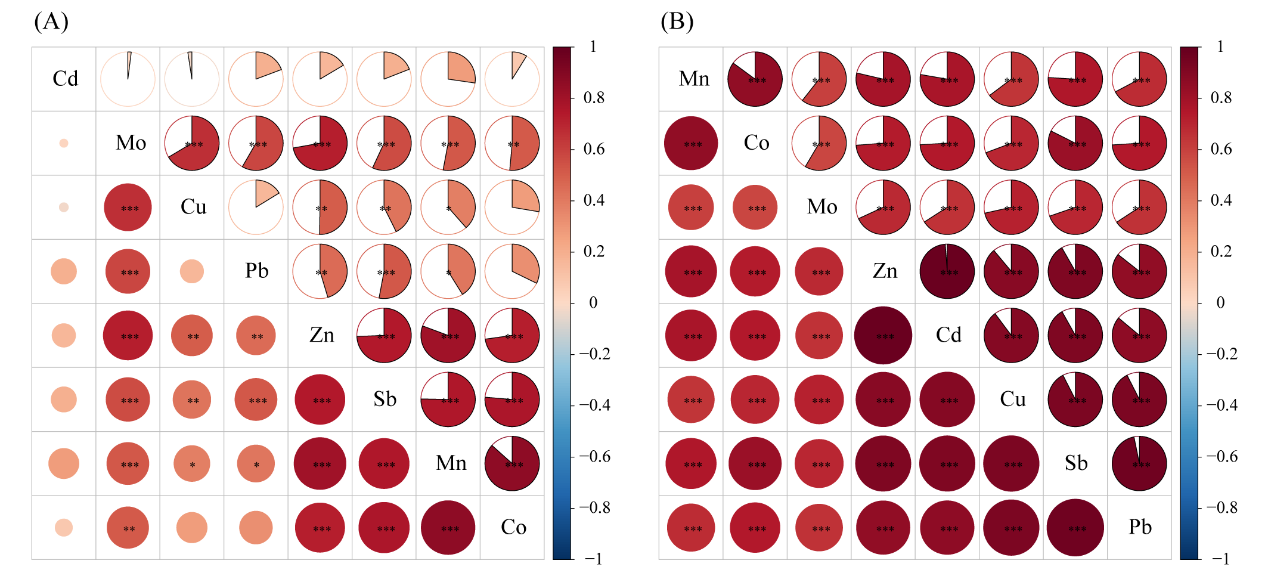


**Supplementary Figure 2. Stability analysis of bacterial co-occurrence networks in the oral buccal mucosa.**

The robustness and vulnerability of the microbial networks in the heavy metal uncontaminated (UA) and contaminated areas (CA). were assessed to compare their resilience to perturbation. (A) Robustness: The relative size of the largest connected component (LCC) is plotted against the fraction of nodes removed, simulating both random failure and targeted attack on high-degree nodes. A more gradual decline indicates a more robust network. (B) Vulnerability: The decline in global network efficiency upon removal of individual nodes is shown. Nodes whose removal causes a sharp efficiency drop are identified as critical keystone taxa (indicated by arrows). These analyses collectively demonstrate a difference in topological stability between the two microbial communities.


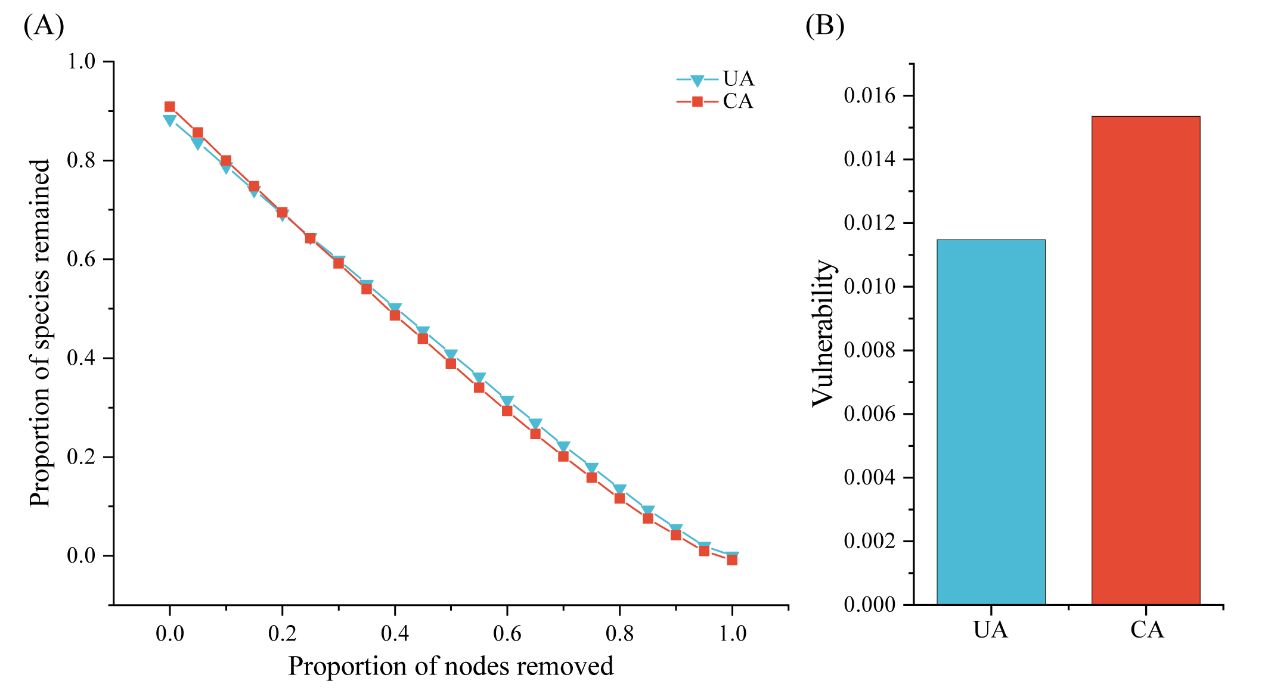


**Supplementary Figure 3. Variability analysis of bacterial KEGG level 2 pathways in oral buccal mucosa**.

The relative abundance of functional pathways between the heavy metal uncontaminated (UA) and contaminated areas (CA) was compared using STAMP software. For each major category, the mean proportion (%) and 95% confidence intervals are shown for (A) Metabolic pathways and (B) Human Disease-related pathways. Only the top 10 pathways exhibiting the most significant differences in mean proportions are displayed. (****P* < 0.001, **0.001 < *P* < 0.01, **P* < 0.05).


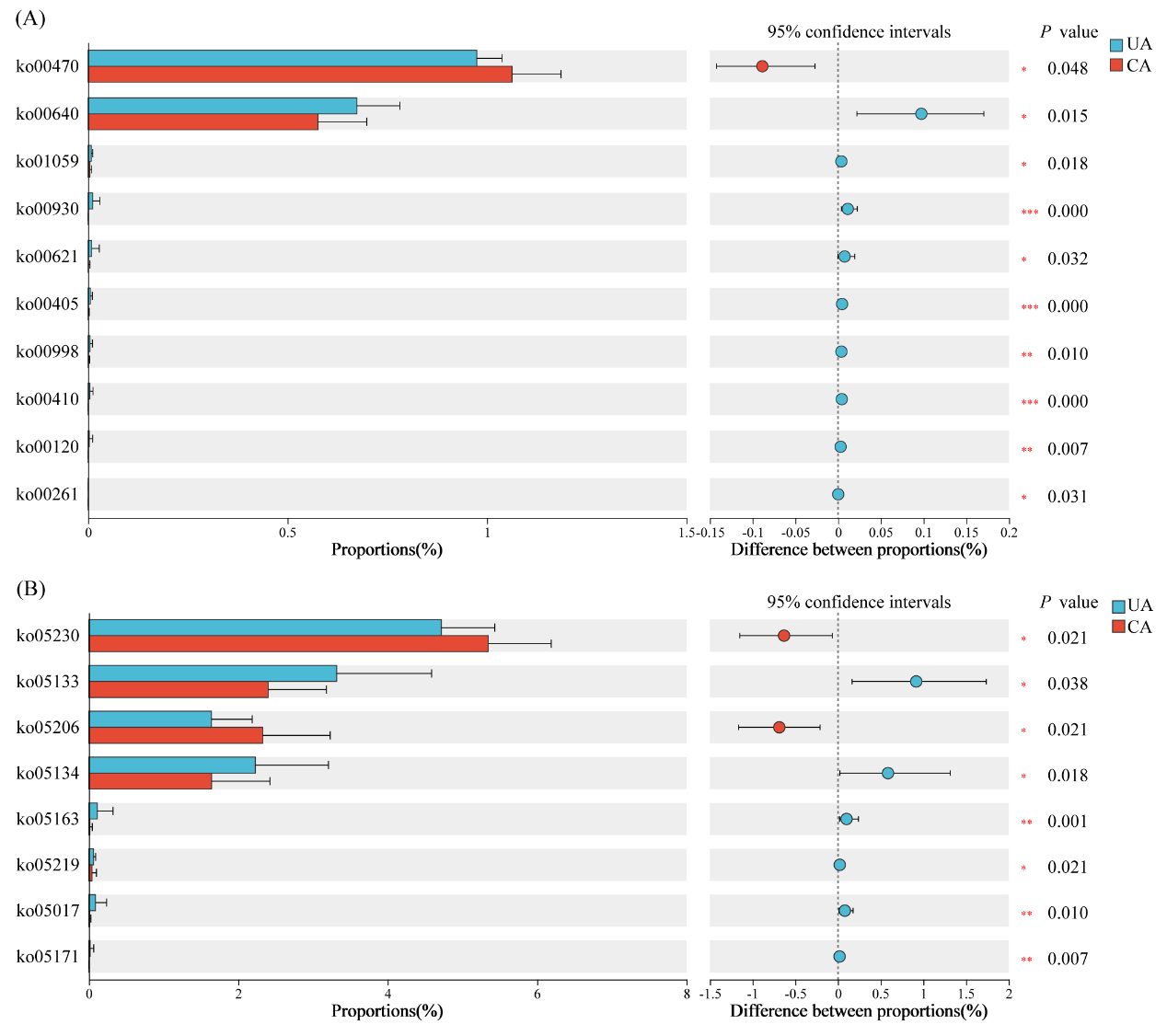


**Supplementary Figure 4. Enriched and depleted KEGG pathways at Level 3 pathways.** Bubble plot displaying the results of functional enrichment analysis comparing the heavy metal uncontaminated (UA) and contaminated areas (CA) for (A) metabolic and (B) human disease-related pathways. Each bubble represents a specific pathway. The horizontal position represents the Reporter Score, indicating the direction and magnitude of pathway alteration (positive scores for enrichment in UA, negative for enrichment in CA). Bubble color corresponds to the Reporter Score value, as shown in the gradient legend. Bubble size is proportional to the statistical significance (-log₁₀(*P*-value)) of the difference. Only pathways meeting a significance threshold (*P* < 0.05) are displayed. This figure identifies specific, fine-grained functional modules that are significantly perturbed in the oral microbiota following heavy metal exposure.


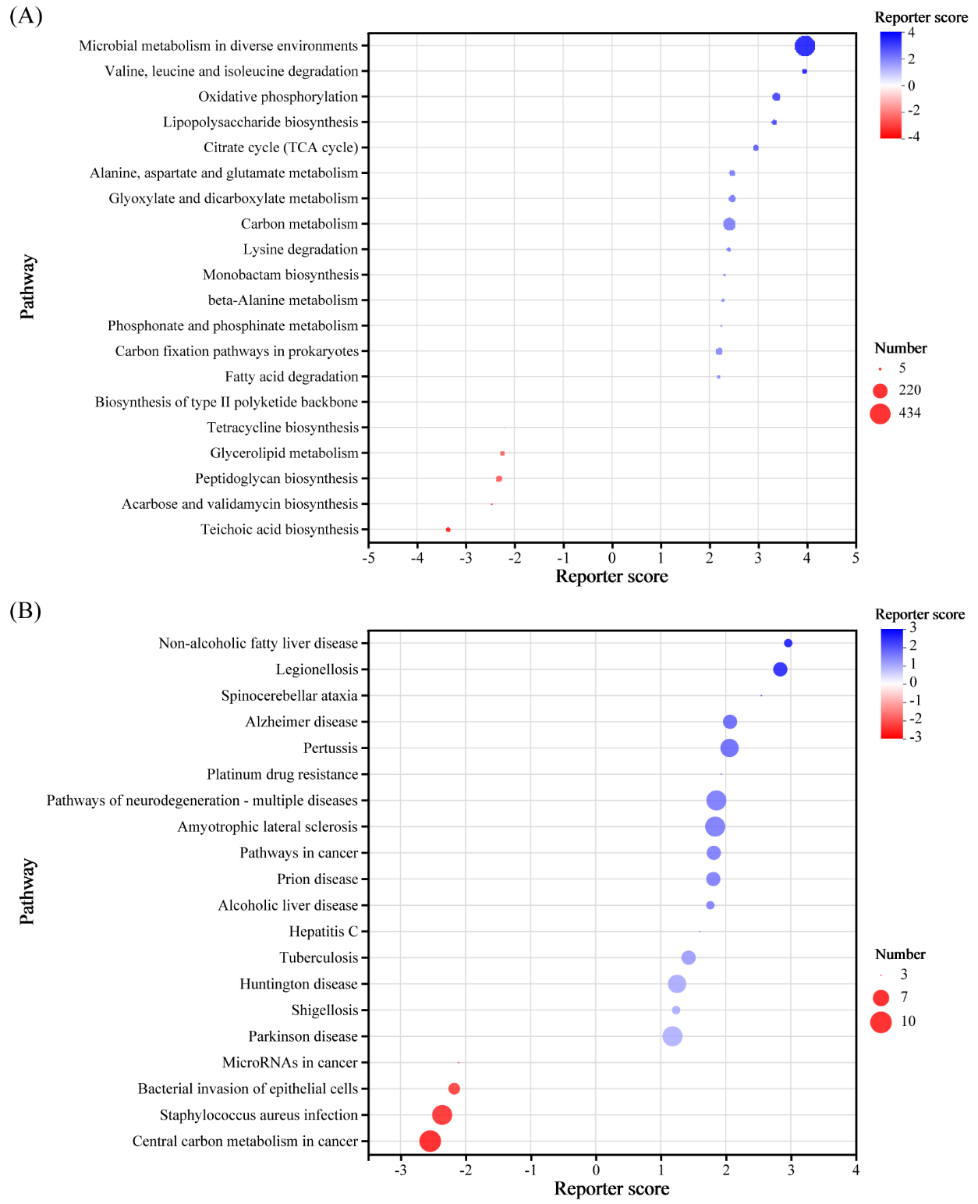


**Supplementary Tables：**

**Supplementary Table 1. Demographic and lifestyle characteristics of the study participants in uncontaminated (UA) and contaminated (CA) areas.**

| Characteristics | UA (n=125) | CA (n=183) | *P-*value |
| --- | --- | --- | --- |
| Age (years) | 55.49±6.74 | 55.92±6.71 | 0.590 |
| BMI (kg/m²) | 23.14±2.94 | 23.10±3.51 | 0.901 |
| Female, n (%) | 76 (60.80%) | 106 (57.92%) | 0.638 |
| Current smoker, n (%) | 97 (77.60%) | 147 (80.33%) | 0.567 |
| Regular drinker, n (%) | 13 (10.40%) | 21 (11.48%) | 0.768 |
| Farming occupation, n (%) | 106 (84.80%) | 158 (86.34%) | 0.705 |

*Note*. Data are presented as mean ± standard deviation (SD) for continuous variables and as number (percentage) for categorical variables. Differences between groups were assessed using independent two-sample t-tests for normally distributed continuous variables and chi-square tests for categorical variables.

**Supplementary Table 2. Demographic and lifestyle characteristics of participants from uncontaminated areas (UA) and contaminated areas (CA) for oral microbiome analysis.**

| Characteristics | UA (n=11) | CA (n=26) | *P-*value |
| --- | --- | --- | --- |
| Age (years) | 55.77(52.33,58.17) | 55.45 (53.29,59.43) | 0.987 |
| BMI (kg/m²) | 22.96 (21.51,26.01) | 23.32 (21.71,25.40) | 0.887 |
| Female, n (%) | 6 (54.55%) | 17 (65.38%) | 0.713 |
| Current smoker, n (%) | 0 (0.00%) | 0 (0.00%) | 1.000 |
| Regular drinker, n (%) | 0 (0.00%) | 0 (0.00%) | 1.000 |
| Farming occupation, n (%) | 11(100.00%) | 26 (100.00%) | 1.000 |

*Note*. Continuous variables, which deviated from a normal distribution, are presented as median and interquartile range (IQR). Categorical variables are presented as number (percentage). Between-group differences were assessed using the Mann-Whitney U test for continuous variables and the chi-square test or Fisher’s exact test for categorical variables.

**Supplementary Table 3. Taxonomic annotation of keyston taxa in the oral bacterial co-occurrence network of the heavy metal uncontaminated (UA) and contaminated areas (CA).**

| Group | Species | Phylum |
| --- | --- | --- |
| UA | *Cutibacterium acnes* | Actinobacteria |
|  | *Peptostreptococcus sp.* | Firmicutes |
|  | *Streptococcus xiaochunlingii* | Firmicutes |
|  | *Fibrobacter sp.* | Fibrobacteres |
|  | *Filifactor alocis* | Firmicutes |
|  | *Neisseria sp. HMSC061B04* | Proteobacteria |
|  | *Neisseria sp. HMSC066H01* | Proteobacteria |
|  | *Porphyromonas macacae* | Bacteroidota |
|  | *Prevotella koreensis* | Bacteroidota |
|  | *Prevotella sp. KH2C16* | Bacteroidota |
|  | *Veillonella dispar* | Firmicutes |
|  | *Veillonella sp.* | Firmicutes |
| CA | *Burkholderia mallei* | Proteobacteria |

*Note*. The table presents the identified keystone taxa separately for the UA and CA, alongside their corresponding Phylum-level classification. This presentation allows for a direct comparison of the identity and broad taxonomic affiliation of the most structurally critical species within the microbial communities under contrasting environmental conditions.

**Supplementary Table 4. Level 2 pathways of major differential genes in oral bacteria in the control group compared to the exposure group.**

(only the top 5 genes in terms of change are shown in the table).

| Name of gene | Abundance | Level 2 pathways |
| --- | --- | --- |
| *gumG* | upregulate | ko00543 |
| *asrC* |  | ko00920, ko01120 |
| *E3.5.4.16* |  | ko00790, ko01240 |
| *glmE, mutE, mamB* |  | ko01120, ko00660, ko00630, ko01200 |
| *ATPVG, ahaH, atpH* |  | ko00190 |
| *POP2* | downregulate | ko01120, ko00650, ko00250 |
| *fadN* |  | ko00362, ko01120, ko01212, ko00650, ko01200, ko00071 |
| *DCAA* |  | ko00920, ko01120 |
| *cobS* |  | ko00860, ko01240 |
| *E6.4.1.4A* |  | ko00280 |

*Note.* This table lists the top 5 genes exhibiting the greatest magnitude of change in abundance between the heavy metal uncontaminated (UA) and contaminated areas (CA). For each entry, the ‘Abundance’ column indicates whether the gene is upregulated or downregulated in the UA group relative to the CA group. The ‘Level 2 pathways’ column provides the corresponding KEGG Orthology (KO) identifiers for the broad functional pathways to which these genes are annotated.

**Supplementary Table 5. Significant correlations between oral bacterial genera and internal biomarkers.**

| Oral Bacterial Genus | Biomarker | Pearson’s R | FDR-adjusted P (*q*-value) ¹ | Direction |
| --- | --- | --- | --- | --- |
| *Actinomyces* | Urine Cd | -0.5785 | 0.0015 | negtive |
| *Gemella* | Urine Cd | 0.54022 | 0.0034 | positive |
| *Streptococcus* | Urine Cd | 0.4926 | 0.0084 | positive |
| *Neisseria* | TNF-ɑ | 0.4581 | 0.01506 | positive |
| *Streptococcus* | TNF-ɑ | -0.4362 | 0.0212 | negtive |
| *Neisseria* | SII | 0.3787 | 0.0477 | positive |

¹ *P*-values were adjusted for multiple testing using the Benjamini–Hochberg false discovery rate (FDR) procedure. This table lists associations with an FDR *q*-value < 0.05, sorted in descending order by the absolute value of Pearson’s R.
